# Wave propagation in a generalized magneto-micropolar thermoelastic medium with gravity and initial stress

**DOI:** 10.1038/s41598-026-49576-y

**Published:** 2026-05-14

**Authors:** Doaa. M. Salah, A. M. Abd-Alla, Mashael A. Aljohani

**Affiliations:** 1 Department of Mathematics, Faculty of Science, Sohag University, Sohag, Egypt; 2 Department of Mathematics and Statistics, College of Science in Yanbu, Taibah University, Yanbu Governorate, Saudi Arabia

**Keywords:** Gravity field, Initial stress, Magneto-micropolar medium, Generalized thermoelasticity, Normal mode analysis, Engineering, Materials science, Mathematics and computing, Physics

## Abstract

This study investigates elastic wave propagation in a generalized magneto-micropolar thermoelastic medium under the combined effects of gravity, initial stress, and a magnetic field. The governing equations, based on the micropolar thermoelastic framework, account for micro-rotation, thermal conduction, electromagnetic interactions, and initial stress, providing a comprehensive description of the medium’s behavior. Analytical expressions for displacement, stress components, and microrotation fields are obtained using the normal mode method, while numerical simulations in Mathematica illustrate how time, magnetic field, gravity, and initial stress influence wave speed, dispersion, and attenuation. The results show that magnetic and micropolar properties reshape wave behavior and introduce distinct dispersive and damping patterns. These findings highlight the complex interplay of multi-physical effects and offer insights for optimizing wave control in microstructured and multifunctional materials, with potential applications in materials science, geophysics, and advanced engineering systems.

## Introduction

Elastic wave propagation in complex thermoelastic media plays a vital role in many scientific and engineering applications, including seismic analysis, nondestructive testing, smart material design, and microscale structural systems. In such environments, wave characteristics are strongly governed by the interaction between mechanical deformation, thermal effects, and additional physical fields. A rigorous theoretical framework capable of capturing these coupled phenomena is therefore essential for an accurate description of wave motion in advanced materials. Previous studies have extensively examined wave propagation in thermoelastic media; however, most works considered simplified models that neglect the combined influence of microstructure, magnetic fields, gravity, and initial stress. This study addresses this gap by investigating a generalized magneto-micropolar thermoelastic medium, providing a comprehensive understanding of how these factors collectively affect wave behavior. By integrating the effects of gravity and initial stress with magnetic and microstructural interactions, our work presents a more realistic and thorough analysis of wave propagation phenomena in complex materials. The classical theory of thermoelasticity was first formulated by Biot^1^, who introduced irreversible thermodynamics into elastic solids. Although this theory successfully described thermomechanical coupling, it predicted an infinite speed of heat propagation contradicting physical observations. To overcome this drawback, generalized thermoelastic theories were developed. The Lord–Shulman model^2^ introduced a single thermal relaxation time, while the Green–Lindsay theory^3^ incorporated two relaxation times to account for finite thermal wave speeds. Subsequently, Green and Naghdi^4^ proposed a thermoelastic theory without energy dissipation, offering an alternative and physically consistent formulation for high-frequency thermal processes. As research progressed, it became evident that classical and generalized thermoelastic theories were insufficient to describe materials with internal microstructure. This realization led to the development of micropolar thermoelasticity, in which each material point possesses independent microrotation in addition to translational motion. The influence of initial stress and rotation in magneto-thermoelastic media under gravity was previously investigated^5^, highlighting the significant role of pre-stress and external fields on the medium’s response. Further extensions considered wave phenomena in initially stressed and thermodiffusive media with additional complexities, such as double porosity, demonstrating the sensitivity of wave propagation to pre-existing stress and material heterogeneity^6^. The role of initial stress was further examined in thermoelastic media with voids and micro temperatures, where it was shown that microstructural thermal effects substantially alter the dynamic response of the medium^7^. These studies emphasized the importance of incorporating internal length-scale effects and thermal microstructure when modeling advanced materials under pre-stressed conditions. More recently, attention has been directed toward magneto-thermoelastic media and their applications in multifunctional and smart materials. The combined effects of magnetic fields, diffusion, and photothermal interactions were analyzed in semiconductor and porous media, revealing pronounced modifications in wave behavior due to electromagnetic coupling^8^. Memory-dependent and nonlocal effects were also introduced into micropolar thermoelastic models, further enriching the description of wave propagation in complex continua^9,10^. The influence of nonlocality, rotation, diffusion, and double porosity on thermoelastic response was systematically studied^11,12^, confirming their strong impact on wave attenuation and dispersion. The interaction between magnetic fields and elastic waves has also been explored in generalized magneto-thermoelastic materials, particularly in the context of wave reflection and refraction^13^. Rotational effects on wave propagation in magneto-micropolar thermoelastic media were investigated in^14^, demonstrating the crucial role of microrotation in modifying wave characteristics. Photothermal interactions in micropolar generalized thermoelastic media subjected to electromagnetic fields were analyzed in^15^, further extending the applicability of magneto-micropolar theories.

The combined influence of gravity, magnetic fields, and generalized thermal effects has been examined in several studies. Reflection of elastic waves in magneto-micropolar thermoelastic media under gravity and two-temperature theory was discussed^16^, while the effects of initial stress and gravity on micropolar thermoelastic solids with micro temperatures were analyzed^17^. Additional investigations considered the influence of rotation and gravity on wave reflection in thermo-magneto-microstretch media under initial stress^18^, as well as the role of initial stress in generalized magneto-thermoelasticity with two-temperature theory^19^. Thermomechanical deformation in orthotropic micropolar thermoelastic solids was finally addressed in^20^, highlighting the versatility of micropolar frameworks in modeling anisotropic and microstructured materials. Micropolar generalized magneto-thermoelasticity has been previously studied to account for modified Ohm’s and Fourier’s laws, highlighting the role of micro-rotations and couple stresses in wave propagation^21^. Recent studies have explored wave propagation and thermoelastic behavior in generalized, nonlocal, and fractional-order semiconductor and porous media under the influence of thermal, magnetic, rotational, and initial stress effects^22–28^. Further studies investigated wave propagation in microstretch, micropolar, and semiconductor thermoelastic media considering rotation, variable thermal conductivity, Hall currents, and photothermal excitation processes^29–33^. However, the combined influence of gravity, initial stress, and magnetic field within a generalized magneto-micropolar thermoelastic framework remains insufficiently explored, motivating the present study. Recent studies have investigated wave reflection and transmission phenomena in photo-piezo, piezo-hygro-thermo-elastic, and nonlocal piezo-thermoelastic media under various higher-order, fractional, and dual-temperature models^34–37^, highlighting the influence of microstructural, pre-stressed, and memory-dependent effects on wave propagation characteristics. Unlike earlier studies on micropolar thermoelastic media with gradually increasing internal heat generation, the present work adopts a more generalized framework by incorporating the combined effects of gravity, initial stress, and magnetic field. While previous studies have explored magneto-thermoelastic wave propagation, the present work extends these analyses by simultaneously considering magnetic field, gravity, and initial stress in a generalized magneto-micropolar thermoelastic medium. This approach provides new insights into the coupled effects on wave stability, attenuation, and dispersion, which have not been comprehensively addressed in earlier research^5,8,11–15^.

Despite the substantial body of literature on magneto-thermoelastic wave propagation, the coupled influence of gravity, initial stress, and magnetic field within the framework of generalized magneto-micropolar thermoelasticity remains insufficiently explored. Addressing this gap, the present study develops a comprehensive theoretical model that accounts for microrotation, thermal effects, magnetic field interactions, gravitational forces, and pre-existing stress. The governing equations are formulated and solved analytically using the normal mode technique, yielding closed-form expressions for the temperature, thermal stress, displacement, and microrotation fields. In addition to obtaining exact solutions, the present work emphasizes the qualitative analysis of wave propagation behavior, providing detailed insight into the stability, attenuation, and dispersion characteristics of the medium under the influence of coupled physical parameters. The results clarify how the interaction between magnetic field, gravity, initial stress, and micropolar effects governs the propagation and damping of thermoelastic waves, thereby advancing the understanding of complex heat conduction mechanisms beyond classical and previously studied models, particularly in the presence of coupled multiphysical effects.

Numerical simulations carried out in Mathematica are presented to support the qualitative analysis and to elucidate the individual and combined effects of time, magnetic field intensity, gravity, and initial stress on wave propagation characteristics, including wave speed, dispersion, and attenuation. The findings of this work provide valuable physical insight and are expected to support the design and optimization of advanced functional materials and engineering systems operating under electromagnetic coupling, pre-stressed conditions, and gravitational environments, with potential applications in geophysics, aerospace engineering, and microscale technologies. The motivation behind the present formulation lies in the need for a more realistic theoretical framework capable of capturing the combined effects of magnetic field, gravity, and initial stress, which are essential in many practical engineering and geophysical applications.

## Basic equations

The behavior of the electromagnetic fields within the considered medium is governed by Maxwell’s equations, which describe the interaction between the electric and magnetic fields and the induced current density. These equations are expressed as

1 $$\:\mathrm{c}\mathrm{u}\mathrm{r}\mathrm{l}\:\mathrm{h}=\mathrm{J}+\dot{D},$$

2 $$\:\mathrm{c}\mathrm{u}\mathrm{r}\mathrm{l}\:\mathrm{E}=-\dot{B},$$

3 $$\:div\:\mathrm{B}=0,\:div\:\mathrm{D}=\rho\:{c}_{e},$$

4 $$\:\mathrm{B}={\mu\:}_{o}H,\:\mathrm{D}={\epsilon\:}_{o}E,$$

Where $\:H{={{\rm\:H}}}_{o}+h$, with $\:{{{\rm\:H}}}_{o}$​ denoting the externally applied magnetic field and $\:h$ representing the induced perturbation of the total magnetic field. The superposed dot denotes differentiation with respect to time. To account for the finite electrical conductivity of the medium and the coupling between the electromagnetic and thermal fields, Maxwell’s equations are supplemented by a modified form of Ohm’s law, given by

5 $$\:J={\sigma\:}_{o}\left[E+\dot{u}\times\:B\right]+{\rho\:}_{e}\dot{u}-{k}_{o}grad\theta\:,$$

where $\:{\sigma\:}_{o}$​ is the electrical conductivity, $\:u$ denotes the displacement vector, $\:\theta\:$ is the temperature increment, and $\:{k}_{o}$​ is the coefficient characterizing the coupling between the temperature gradient and the electric current density. The linear momentum balance equation, accounting for the Lorentz force and in the absence of body forces, can be expressed as:

6 $$\:{\sigma\:}_{ji,j}+{(J\times\:B)}_{i}+{\rho\:}_{e}{E}_{i}=\rho\:\ddot{u},$$

where the comma denotes differentiation with respect to the spatial coordinates. The angular momentum balance equation, in the absence of body couples and considering the electromagnetic couples, is given by:

7 $$\:{e}_{ijk}{\sigma\:}_{jk}+{\mu\:}_{ji,j}+{e}_{ijk}{\chi\:}_{j}{(J\times\:B)}_{k}=\rho\:j{\omega\:}_{i},$$

The energy equation, in the absence of a heat source, takes the form:

8 $$\:\rho\:{T}_{o}\dot{\eta\:}=-{q}_{i,i},$$

In an isotropic medium, the entropy $\:\eta\:$ can be expressed in terms of temperature as:

9 $$\:\rho\:\eta\:=\frac{\rho\:{c}_{e}\theta\:}{{T}_{o}}+\gamma\:e,$$

The generalized Fourier’s law, accounting for the influence of current density, can be expressed as:

10 $$\:{q}_{i}+{\tau\:}_{o}\dot{{q}_{i}}=-k{\theta\:}_{,i}+{\pi\:}_{o}{J}_{i},$$

where $\:{\pi\:}_{o}$​ is the coefficient that relates the current density to the heat flux. Within the framework of linear theory, all second-order and higher-order terms are neglected. Consequently, the governing Eqs. (5)– (8) can be represented in the following linearized form:

11 $$\:J={\sigma\:}_{o}\left[E+{\mu\:}_{o}\dot{u}\times\:{{{\rm\:H}}}_{o}\right]-{k}_{o}grad\theta\:,$$

12 $$\:{\sigma\:}_{ji,j}+{\mu\:}_{o}{(J\times\:{{{\rm\:H}}}_{o})}_{i}=\rho\:\ddot{u},$$

13 $$\:{e}_{ijk}{\sigma\:}_{jk}+{\mu\:}_{ji,j}=\rho\:j{w}_{i},$$

14 $$\:\rho\:{T}_{o}\dot{\eta\:}=-{q}_{i,i},$$

Eliminating $\:\eta\:$ between Eqs. (9) and (14), and incorporating Eq. (10), the heat conduction equation within the framework of linear theory can be expressed as follows:

15 $$\:k{\nabla\:}^{2}\theta\:=\left(\frac{\partial\:}{\partial\:t}+{\tau\:}_{o}\frac{{\partial\:}^{2}}{\partial\:{t}^{2}}\right)(\rho\:{c}_{e}\theta\:+\gamma\:{T}_{o}e+{\pi\:}_{o}div\:\dot{J},$$

Equations (1)– (4), (11)– (13) and (15) are supplemented by the following constitutive relations

16 $$\:{\sigma\:}_{ij}=\lambda\:{u}_{k,k}{\delta\:}_{ij}+(\mu\:+\alpha\:){u}_{j,i}+(\mu\:-\alpha\:){u}_{i,j}-2\alpha\:{e}_{ijk}{\omega\:}_{k}-\gamma\:\theta\:{\delta\:}_{ij},$$

17 $$\:{\mu\:}_{ij}=\epsilon\:{\omega\:}_{k,k}{\delta\:}_{ij}+(\nu\:+\beta\:){\omega\:}_{i,j}+(\nu\:-\beta\:){\omega\:}_{i,j},$$

18 $$\:e=div\:u.$$

Therefore, Eqs. (1)– (4), (11)– (13), and (15)– (18) together form the set of field equations and constitutive relations governing the linear theory of micropolar generalized magneto-thermoelasticity, incorporating the modified Ohm’s law and the generalized Fourier’s law.

## Formulation of the problem

**Fig. 1:**
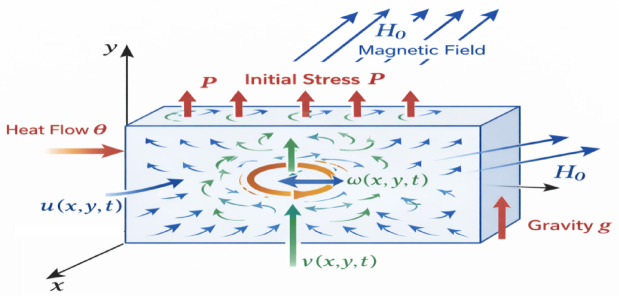
Problem geometry.

We consider a homogeneous, isotropic generalized magneto-micropolar thermoelastic medium occupying a two-dimensional Cartesian coordinate system $\:(x,y)$. The medium is assumed to be initially stressed and subjected to a uniform gravitational field, as well as an external magnetic field. The analysis is carried out within the framework of linear micropolar thermoelasticity. Let $\:u(x,y,t)$ and $\:v(x,y,t)$ denote the displacement components along the $\:x\:$and$\:\:y\:$directions, respectively, and let $\:\omega\:(x,y,t)$ represent the microrotation about the out-of-plane axis. The governing equations are established in line with the methodology of Ezzat and Awad^21^, who extended classical micropolar theory by employing modified Ohm’s and Fourier’s laws to account for coupled electromagnetic and thermal effects in microstructured media (Fig. 1).

The equations of motion are described as follows:

19 $$\begin{aligned}\:\left(\lambda\:+2\mu\:+p\right)\left(\frac{{\partial\:}^{2}u}{\partial\:{x}^{2}}+\frac{{\partial\:}^{2}v}{\partial\:x\partial\:y}\right)&+\left(\mu\:+\alpha\:+p\right)\left(\frac{{\partial\:}^{2}u}{\partial\:{y}^{2}}-\frac{{\partial\:}^{2}v}{\partial\:x\partial\:y}\right)+{\sigma\:}_{o}{\mu\:}_{o}{{{\rm\:H}}}_{o}{{{\rm\:E}}}_{2}\\&+2\alpha\:\frac{\partial\:\omega\:}{\partial\:y}-\gamma\:(\frac{\partial\:\theta\:}{\partial\:x}+{k}_{o}\frac{\partial\:\theta\:}{\partial\:y})-\rho\:g\frac{\partial\:v}{\partial\:x}=\rho\:(\frac{{\partial\:}^{2}u}{\partial\:{t}^{2}}+{\sigma\:}_{o}{\mu\:}_{o}{{{\rm\:H}}}_{o}\frac{\partial\:u}{\partial\:t}),\end{aligned}$$

20 $$\begin{aligned}\:\left(\lambda\:+2\mu\:+p\right)\left(\frac{{\partial\:}^{2}v}{\partial\:{y}^{2}}+\frac{{\partial\:}^{2}u}{\partial\:x\partial\:y}\right)&+\left(\mu\:+\alpha\:+p\right)\left(\frac{{\partial\:}^{2}v}{\partial\:{x}^{2}}-\frac{{\partial\:}^{2}u}{\partial\:x\partial\:y}\right)-{\sigma\:}_{o}{\mu\:}_{o}{{{\rm\:H}}}_{o}{{{\rm\:E}}}_{1}-2\alpha\:\frac{\partial\:\omega\:}{\partial\:x}\\&-\gamma\:(\frac{\partial\:\theta\:}{\partial\:x}-{k}_{o}\frac{\partial\:\theta\:}{\partial\:y})-\rho\:g\frac{\partial\:u}{\partial\:x}=\rho\:(\frac{{\partial\:}^{2}v}{\partial\:{t}^{2}}+{\sigma\:}_{o}{\mu\:}_{o}{{{\rm\:H}}}_{o}\frac{\partial\:v}{\partial\:t}),\end{aligned}$$

The heat conduction equation is expressed as:

21 $$\:k(\frac{{\partial\:}^{2}\theta\:}{\partial\:{x}^{2}}+\frac{{\partial\:}^{2}\theta\:}{\partial\:{y}^{2}})=\left(\frac{\partial\:}{\partial\:t}+{\tau\:}_{o}\frac{{\partial\:}^{2}}{\partial\:{t}^{2}}\right)(\rho\:{c}_{e}\theta\:+\gamma\:{T}_{o}\left(\frac{\partial\:u}{\partial\:x}+\frac{\partial\:v}{\partial\:y}\right)-{\pi\:}_{o}{\sigma\:}_{o}\frac{\partial\:}{\partial\:t}(\frac{\partial\:{{{\rm\:E}}}_{1}}{\partial\:x}+\frac{\partial\:{{{\rm\:E}}}_{2}}{\partial\:y}),$$

The micropolar equations can be formulated as:

22 $$\:(\upsilon\:+\beta\:)\left(\frac{{\partial\:}^{2}}{\partial\:{x}^{2}}+\frac{{\partial\:}^{2}}{\partial\:{y}^{2}}\right)\omega\:-2\alpha\:\left(\frac{\partial\:u}{\partial\:y}-\frac{\partial\:v}{\partial\:x}\right)-4\alpha\:\omega\:=\rho\:j\frac{{\partial\:}^{2}\omega\:}{\partial\:{t}^{2}},$$

The stress components are

23 $$\:{\sigma\:}_{xx}=(\lambda\:+2\mu\:+p)\frac{\partial\:u}{\partial\:x}+\lambda\:\frac{\partial\:v}{\partial\:y}-\gamma\:\theta\:,$$

24 $$\:{\sigma\:}_{yy}=(\lambda\:+2\mu\:+p)\frac{\partial\:v}{\partial\:y}+\lambda\:\frac{\partial\:u}{\partial\:x}-\gamma\:\theta\:,$$

25 $$\:{\sigma\:}_{zz}=\lambda\:\left(\frac{\partial\:u}{\partial\:x}+\frac{\partial\:v}{\partial\:y}\right)-\gamma\:\theta\:,$$

26 $$\:{\sigma\:}_{xy}=\left(\mu\:+\alpha\:\right)\frac{\partial\:v}{\partial\:x}+\left(\mu\:-\alpha\:\right)\frac{\partial\:u}{\partial\:y}-2\alpha\:\omega\:,$$

27 $$\:{\sigma\:}_{yx}=\left(\mu\:+\alpha\:\right)\frac{\partial\:u}{\partial\:y}+\left(\mu\:-\alpha\:\right)\frac{\partial\:v}{\partial\:x}+2\alpha\:\omega\:,$$

28 $$\:{\mu\:}_{xz}=(\upsilon\:+\beta\:)\frac{\partial\:\omega\:}{\partial\:x},$$

29 $$\:{\mu\:}_{zx}=(\upsilon\:-\beta\:)\frac{\partial\:\omega\:}{\partial\:x},$$

30 $$\:{\mu\:}_{yz}=(\upsilon\:+\beta\:)\frac{\partial\:\omega\:}{\partial\:y},$$

31 $$\:{\mu\:}_{zy}=(\upsilon\:-\beta\:)\frac{\partial\:\omega\:}{\partial\:y},$$

32 $$\:\frac{\partial\:h}{\partial\:y}={\sigma\:}_{o}\left[{{{\rm\:E}}}_{1}+{\mu\:}_{o}{{{\rm\:H}}}_{o}\frac{\partial\:v}{\partial\:t}\right]+{\epsilon\:}_{o}\frac{\partial\:{{{\rm\:E}}}_{1}}{\partial\:t}-{k}_{o}\frac{\partial\:\theta\:}{\partial\:x},$$

33 $$\:\frac{\partial\:h}{\partial\:x}={-\sigma\:}_{o}\left[{{{\rm\:E}}}_{2}-{\mu\:}_{o}{{{\rm\:H}}}_{o}\frac{\partial\:u}{\partial\:t}\right]-{\epsilon\:}_{o}\frac{\partial\:{{{\rm\:E}}}_{2}}{\partial\:t}+{k}_{o}\frac{\partial\:\theta\:}{\partial\:y},$$

34 $$\:\frac{\partial\:{E}_{1}}{\partial\:y}-\frac{\partial\:{E}_{2}}{\partial\:x}={\mu\:}_{o}\frac{\partial\:h}{\partial\:t},$$

35 $$\:{J}_{1}={\sigma\:}_{o}\left[{{{\rm\:E}}}_{1}+{\mu\:}_{o}{{{\rm\:H}}}_{o}\frac{\partial\:v}{\partial\:t}\right]-{k}_{o}\frac{\partial\:\theta\:}{\partial\:x},$$

36 $$\:{J}_{2}={\sigma\:}_{o}\left[{{{\rm\:E}}}_{2}-{\mu\:}_{o}{{{\rm\:H}}}_{o}\frac{\partial\:u}{\partial\:t}\right]-{k}_{o}\frac{\partial\:\theta\:}{\partial\:y},$$

To enable efficient numerical computations, the following nondimensional parameters are introduced:

$$\:\left(\stackrel{\prime}{x},\stackrel{\prime}{y},\stackrel{\prime}{u},\stackrel{\prime}{v}\right)={c}_{o}{\eta\:}_{o}\:\left(x,y,u,v\right),\:\left({t}^{{\prime\:}},{{\tau\:}_{o}}^{{\prime\:}}\right)=\:{c}_{o}^{2}{\eta\:}_{o}\left(t,{\tau\:}_{o}\right),\:{\omega\:}^{{\prime\:}}=\frac{\alpha\:}{\mu\:+\alpha\:}\omega\:,\:{\:E}_{i}^{{\prime\:}}=\frac{{\eta\:}_{o}}{{\sigma\:}_{o}{\mu\:}_{o}^{2}{{{\rm\:H}}}_{o}{c}_{o}\:}{E}_{i},$$

$$\:{\:(\sigma\:}_{ij}^{{\prime\:}},\:{p}^{{\prime\:}})=\frac{{(\sigma\:}_{ij},p)}{\mu\:+\alpha\:\:},\:{\theta\:}^{{\prime\:}}=\frac{\gamma\:\theta\:}{\rho\:{c}_{o}^{2}},\:{\:\mu\:}_{ij}^{{\prime\:}}=\frac{\alpha\:{\mu\:}_{ij}}{{c}_{o}{\eta\:}_{o}\left(\mu\:+\alpha\:\right)\left(\upsilon\:+\beta\:\right)},\:{h}^{{\prime\:}}=\frac{{\eta\:}_{o}}{{\sigma\:}_{o}{\mu\:}_{o}{{{\rm\:H}}}_{o}\:}h,\:{g}^{{\prime\:}}=\frac{kg}{\rho\:{c}_{e}{c}_{^\circ\:}^{3}},$$

37 $$\:{{J}_{i}}^{{\prime\:}}=\frac{{\eta\:}_{o}{J}_{i}}{{\sigma\:}_{o}^{2}{\mu\:}_{o}^{2}{{{\rm\:H}}}_{o}{c}_{o}\:}h,\:{c}_{o}^{2}=\frac{\lambda\:+2\mu\:+p}{\rho\:},\:{\eta\:}_{o}=\frac{\rho\:{c}_{e}}{k}.\:$$

Substituting Eq. (38) into Eqs. (19)– (37), and omitting the prime notation for simplicity, we obtain:

38 $$\:{\beta\:}_{1}^{2}\frac{\partial\:e}{\partial\:x}+\frac{\partial\:\phi\:}{\partial\:y}+2\frac{\partial\:\omega\:}{\partial\:y}+{{\beta\:}_{1}^{2}{\upsilon\:}_{1}^{2}{\epsilon\:}_{2}{{\rm\:E}}}_{2}-{\beta\:}_{1}^{2}(\frac{\partial\:\theta\:}{\partial\:x}+{k}_{1}\frac{\partial\:\theta\:}{\partial\:y})-{\upsilon\:}_{2}\frac{\partial\:v}{\partial\:x}={\beta\:}_{1}^{2}(\frac{{\partial\:}^{2}u}{\partial\:{t}^{2}}+{\upsilon\:}_{1}{\epsilon\:}_{2}\frac{\partial\:u}{\partial\:t}),$$

39 $$\:{\beta\:}_{1}^{2}\frac{\partial\:e}{\partial\:y}-\frac{\partial\:\phi\:}{\partial\:x}-2\frac{\partial\:\omega\:}{\partial\:x}-{{\beta\:}_{1}^{2}{\upsilon\:}_{1}^{2}{\epsilon\:}_{2}{{\rm\:E}}}_{1}-{\beta\:}_{1}^{2}\left(\frac{\partial\:\theta\:}{\partial\:x}-{k}_{1}\frac{\partial\:\theta\:}{\partial\:y}\right)+{\upsilon\:}_{2}\frac{\partial\:u}{\partial\:x}={\beta\:}_{1}^{2}(\frac{{\partial\:}^{2}v}{\partial\:{t}^{2}}+{\upsilon\:}_{1}{\epsilon\:}_{2}\frac{\partial\:v}{\partial\:t}),$$

40 $$\:(\frac{{\partial\:}^{2}\theta\:}{\partial\:{x}^{2}}+\frac{{\partial\:}^{2}\theta\:}{\partial\:{y}^{2}})=\left(\frac{\partial\:}{\partial\:t}+{\tau\:}_{o}\frac{{\partial\:}^{2}}{\partial\:{t}^{2}}\right)(\theta\:+{\epsilon\:}_{1}e)-{\pi\:}_{1}\frac{\partial\:}{\partial\:t}(\frac{\partial\:{{{\rm\:E}}}_{1}}{\partial\:x}+\frac{\partial\:{{{\rm\:E}}}_{2}}{\partial\:y}),$$

41 $$\:\left(\frac{{\partial\:}^{2}}{\partial\:{x}^{2}}+\frac{{\partial\:}^{2}}{\partial\:{y}^{2}}\right)\omega\:-{g}_{1}\phi\:-{g}_{2}\omega\:={g}_{3}\frac{{\partial\:}^{2}\omega\:}{\partial\:{t}^{2}},$$

42 $$\:{\sigma\:}_{xx}={\beta\:}_{1}^{2}(\frac{\partial\:u}{\partial\:x}-\theta\:)+{\delta\:}_{1}\frac{\partial\:v}{\partial\:y},$$

43 $$\:{\sigma\:}_{yy}={\beta\:}_{1}^{2}(\frac{\partial\:v}{\partial\:y}-\theta\:)+{\delta\:}_{1}\frac{\partial\:u}{\partial\:x},$$

44 $$\:{\sigma\:}_{zz}={\delta\:}_{1}e-{\beta\:}_{1}^{2}\theta\:,$$

45 $$\:{\sigma\:}_{xy}=\frac{\partial\:v}{\partial\:x}+{\delta\:}_{2}\frac{\partial\:u}{\partial\:y}-2\omega\:,$$

46 $$\:{\sigma\:}_{yx}=\frac{\partial\:u}{\partial\:y}+{\delta\:}_{2}\frac{\partial\:v}{\partial\:x}-2\omega\:,$$

47 $$\:{\mu\:}_{xz}=\frac{\partial\:\omega\:}{\partial\:x},$$

48 $$\:{\mu\:}_{zx}={\delta\:}_{3}\frac{\partial\:\omega\:}{\partial\:x},$$

49 $$\:{\mu\:}_{yz}=\frac{\partial\:\omega\:}{\partial\:y},$$

50 $$\:{\mu\:}_{zy}={\delta\:}_{3}\frac{\partial\:\omega\:}{\partial\:y},$$

51 $$\:\frac{\partial\:h}{\partial\:y}={{\upsilon\:}_{1}{{\rm\:E}}}_{1}+\frac{\partial\:v}{\partial\:t}+{V}^{2}\frac{\partial\:{{{\rm\:E}}}_{1}}{\partial\:t}-{k}_{2}\frac{\partial\:\theta\:}{\partial\:x},$$

52 $$\:\frac{\partial\:h}{\partial\:x}={-{\upsilon\:}_{1}{{\rm\:E}}}_{2}+\frac{\partial\:u}{\partial\:t}-{V}^{2}\frac{\partial\:{{{\rm\:E}}}_{2}}{\partial\:t}+{k}_{2}\frac{\partial\:\theta\:}{\partial\:y},$$

53 $$\:\frac{\partial\:{E}_{1}}{\partial\:y}-\frac{\partial\:{E}_{2}}{\partial\:x}=\frac{\partial\:h}{\partial\:t},$$

54 $$\:{J}_{1}={{{\rm\:E}}}_{1}+\frac{1}{{\upsilon\:}_{1}}\frac{\partial\:v}{\partial\:t}-{k}_{3}\frac{\partial\:\theta\:}{\partial\:x},$$

55 $$\:{J}_{2}={{{\rm\:E}}}_{2}-\frac{1}{{\upsilon\:}_{1}}\frac{\partial\:u}{\partial\:t}-{k}_{3}\frac{\partial\:\theta\:}{\partial\:y}$$

Where

$$\:{\beta\:}_{1}^{2}=\frac{\lambda\:+2\mu\:+p}{\mu\:+\alpha\:},\:{\upsilon\:}_{1}=\frac{{\sigma\:}_{o}{\mu\:}_{o}}{{\eta\:}_{o}\:}, \:{\upsilon\:}_{2}=\frac{\rho\:{c}_{^\circ\:}^{2}g}{\mu\:+\alpha\:+p\:}, \:{V}^{2}=\frac{{c}_{o}^{2}}{{c}^{2}\:}, \:{c}^{2}=\frac{1}{{\mu\:}_{o}{\epsilon\:}_{o}\:}, \:{\epsilon\:}_{1}=\frac{{{\gamma\:}^{2}T}_{o}}{\rho\:{c}_{o}^{2}{\eta\:}_{o}k\:}, \:{\epsilon\:}_{2}=\frac{{\mu\:}_{o}{H}_{o}^{2}}{\rho\:{c}_{o}^{2}\:,}$$

$$\:{k}_{1}=\frac{{\mu\:}_{o}{H}_{o}{k}_{o}}{\gamma\:\:},\:{k}_{2}=\frac{{k}_{1}}{{\upsilon\:}_{1}{\epsilon\:}_{2}\:},\:{k}_{3}=\frac{{k}_{2}}{{\upsilon\:}_{1}\:},{\pi\:}_{1}={\pi\:}_{o}^{*}{\epsilon\:}_{2}{V}^{2},\:{\pi\:}_{o}^{*}=\frac{{\sigma\:}_{o}{\pi\:}_{o}\gamma\:}{k{H}_{o}\:},\:{g}_{1}=\frac{2{\alpha\:}^{2}}{{c}_{o}^{2}{\eta\:}_{o}^{2}(\mu\:+\alpha\:+p)(\upsilon\:+\beta\:)\:},$$

$$\:{g}_{2}=\frac{4\alpha\:}{{c}_{o}^{2}{\eta\:}_{o}^{2}(\upsilon\:+\beta\:)\:}, \:{g}_{3}=\frac{\rho\:j{c}_{o}^{2}}{\upsilon\:+\beta\:\:}, \:{\delta\:}_{1}=\frac{\lambda\:}{\mu\:+\alpha\:}, \:{\delta\:}_{2}=\frac{\mu\:-\alpha\:}{\mu\:+\alpha\:},\:{\delta\:}_{3}=\frac{\upsilon\:-\beta\:}{\upsilon\:+\beta\:},\:\:e=\frac{\partial\:u}{\partial\:x}+\frac{\partial\:v}{\partial\:y}, \:\phi\:=\left(\frac{\partial\:u}{\partial\:y}-\frac{\partial\:v}{\partial\:x}\right).$$

By applying suitable spatial differentiations and using the definitions of dilatation and microrotation, the displacement components are eliminated, yielding a reduced system of governing equations in terms of dilatation, microrotation, magnetic field, and temperature. Consequently, this system takes the following form:

56 $$\:\left({\nabla\:}^{2}-\frac{{\partial\:}^{2}}{\partial\:{t}^{2}}-{\upsilon\:}_{1}{\epsilon\:}_{2}\frac{\partial\:}{\partial\:t}\right)e={\nabla\:}^{2}\theta\:-{\upsilon\:}_{3}\frac{\partial\:\phi\:}{\partial\:x}+{\upsilon\:}_{1}^{2}{\epsilon\:}_{2}\frac{\partial\:h}{\partial\:t},$$

57 $$\:\left[{\nabla\:}^{2}-{\beta\:}_{1}^{2}\left(\frac{{\partial\:}^{2}}{\partial\:{t}^{2}}+{\upsilon\:}_{1}{\epsilon\:}_{2}\frac{\partial\:}{\partial\:t}\right)\right]\phi\:={\beta\:}_{1}^{2}{k}_{1}{\nabla\:}^{2}\theta\:+{\upsilon\:}_{2}\frac{\partial\:e}{\partial\:x}-2{\nabla\:}^{2}\omega\:-{\beta\:}_{1}^{2}{\upsilon\:}_{1}^{2}{\epsilon\:}_{2}(\frac{\partial\:{E}_{1}}{\partial\:x}+\frac{\partial\:{E}_{2}}{\partial\:y}),$$

58 $$\:({\nabla\:}^{2}-{V}^{2}\frac{{\partial\:}^{2}}{\partial\:{t}^{2}}-{\upsilon\:}_{1}\frac{\partial\:}{\partial\:t})h=\frac{\partial\:e}{\partial\:t},$$

59 $$\:\left({\upsilon\:}_{1}+{V}^{2}\frac{\partial\:}{\partial\:t}\right)\left[\frac{\partial\:{E}_{1}}{\partial\:x}+\frac{\partial\:{E}_{2}}{\partial\:y}\right]=\frac{\partial\:\phi\:}{\partial\:t}+{k}_{2}{\nabla\:}^{2}\theta\:.$$

## Normal mode analysis

We assume that all field variables admit plane wave solutions and can therefore be represented in the following exponential form:

60 $$\:\left(\mathrm{e},\theta\:,\phi\:\:,\omega\:,h{,\sigma\:}_{ij},{\mu\:}_{ij}\right)\left(x,y,t\right)=\left({\mathrm{e}}^{*},{\theta\:}^{*},{\phi\:}^{*},{\omega\:}^{*},{h}^{*}{{,\sigma\:}_{ij}}^{*}{,{\mu\:}_{ij}}^{*}\right)\left(x\right){e}^{(\varpi\:t+iay)}.$$

Where $\:\varpi\:$ denotes the complex frequency, $\:a$ is the wave number in the $\:y-$direction, $\:i$ is the imaginary unit, and the starred quantities represent the corresponding wave amplitudes. Substituting Eq. (61) into Eqs. (41)– (42) and (57)– (60), the governing equations reduce to the following system:

61 $$\:\left({\mathrm{D}}^{2}-{\eta\:}_{1}\right){e}^{*}-\left({\mathrm{D}}^{2}-{\mathrm{a}}^{2}\right){\theta\:}^{*}+{\upsilon\:}_{3}D{\phi\:}^{*}-{\eta\:}_{2}{h}^{*}=0,$$

62 $$\:{\upsilon\:}_{2}D{e}^{*}+{{\eta\:}_{4}\left({\mathrm{D}}^{2}-{\mathrm{a}}^{2}\right)\theta\:}^{*}-\left({\mathrm{D}}^{2}-{\eta\:}_{3}\right){\phi\:}^{*}-2\left({\mathrm{D}}^{2}-{\mathrm{a}}^{2}\right){\omega\:}^{*}=0,$$

63 $$\:{g}_{1}{\phi\:}^{*}-\left({\mathrm{D}}^{2}-{\eta\:}_{5}\right){\omega\:}^{*}=0,$$

64 $$\:{\eta\:}_{7}{e}^{*}-{\left({\mathrm{D}}^{2}-{\eta\:}_{6}\right)\theta\:}^{*}-{\eta\:}_{8}{\phi\:}^{*}=0,$$

65 $$\:\varpi\:{e}^{*}-\left({\mathrm{D}}^{2}-{\eta\:}_{9}\right){h}^{*}=0.$$

Where

$$\:D=\frac{d}{dx},\:{D}^{2}=\frac{{d}^{2}}{{dx}^{2}},\:{\upsilon\:}_{3}=\frac{{\upsilon\:}_{2}}{{\beta\:}_{1}^{2}},\:{\eta\:}_{1}={\mathrm{a}}^{2}+{\varpi\:}^{2}+{\upsilon\:}_{1}{\epsilon\:}_{2}\varpi\:,\:{\eta\:}_{2}{=\upsilon\:}_{1}^{2}{\epsilon\:}_{2}\varpi\:,$$

$$\:{\eta\:}_{3}{=\mathrm{a}}^{2}+{\beta\:}_{1}^{2}{\varpi\:}^{2}+{\beta\:}_{1}^{2}{\upsilon\:}_{1}{\epsilon\:}_{2}\varpi\:-\frac{{\beta\:}_{1}^{2}{\upsilon\:}_{1}{\epsilon\:}_{2}\varpi\:}{{\upsilon\:}_{1}+{V}^{2}\varpi\:},\:{\eta\:}_{4}={\beta\:}_{1}^{2}\left({k}_{1}-\frac{{\upsilon\:}_{1}^{2}{\epsilon\:}_{2}{k}_{2}}{{\upsilon\:}_{1}+{V}^{2}\varpi\:}\right),$$

$$\:{\eta\:}_{5}={\mathrm{a}}^{2}+{g}_{2}+{g}_{3}\varpi\:,\:{\eta\:}_{6}={\mathrm{a}}^{2}+\frac{\varpi\:\left(1+{\tau\:}_{o}\varpi\:\right)\left({\upsilon\:}_{1}+{V}^{2}\varpi\:\right)}{{\upsilon\:}_{1}+{V}^{2}\varpi\:+{\pi\:}_{1}\varpi\:{k}_{2}},$$

$$\:{\eta\:}_{7}=\frac{{\epsilon\:}_{1}\varpi\:(1+{\tau\:}_{o}\varpi\:)({\upsilon\:}_{1}+{V}^{2}\varpi\:)}{{\upsilon\:}_{1}+{V}^{2}\varpi\:+{\pi\:}_{1}\varpi\:{k}_{2}}\:,\:{\eta\:}_{8}=\frac{{\pi\:}_{1}{\varpi\:}^{2}}{{\upsilon\:}_{1}+{V}^{2}\varpi\:+{\pi\:}_{1}\varpi\:{k}_{2}},\:{\eta\:}_{9}={\mathrm{a}}^{2}+{V}^{2}{\varpi\:}^{2}+{\upsilon\:}_{1}\varpi\:.$$

By solving Eqs. (62)- (66), a tenth-order homogeneous differential equation is obtained in the following form:

66 $$\:[{D}^{10}+{A}_{11}{D}^{8}+{A}_{22}{D}^{7}+{A}_{33}{D}^{6}+{A}_{44}{D}^{5}+{A}_{55}{D}^{4}+{A}_{66}{D}^{3}+{A}_{77}{D}^{2}+{A}_{88}D+{A}_{99}]\{{e}^{*},{\:\theta\:}^{*},{\:\phi\:}^{*},\:{h}^{*},\:{\omega\:}^{*}\}=0.$$

The constants $\:{A}_{11}$, $\:{A}_{22}$, $\:{A}_{33}$, $\:{A}_{44}$, $\:{A}_{55}$, $\:{A}_{66}$, $\:{A}_{77}$, $\:{A}_{88}$, and $\:{A}_{99}$ are defined in Appendix A.

The corresponding characteristic equation associated with Eq. (67) is given by:

67 $$\:{\lambda\:}^{10}+{A}_{11}{\lambda\:}^{8}+{A}_{22}{\lambda\:}^{7}+{A}_{33}{\lambda\:}^{6}+{A}_{44}{\lambda\:}^{5}+{A}_{55}{\lambda\:}^{4}+{A}_{66}{\lambda\:}^{3}+{A}_{77}{\lambda\:}^{2}+{A}_{88}\lambda\:+{A}_{99}=0.$$

Unlike the classical elastic case, the presence of magnetic field and gravity breaks the symmetry of the medium, resulting in odd-order terms in the characteristic equation. These terms play a crucial role in describing wave attenuation and directional effects. Only the roots with positive real parts are retained to ensure bounded solutions. The general solutions for all physical quantities, subject to the condition as *x*→∞, are expressed as follows:

68 $$\:{h}^{*}\left(x\right)=\sum\:_{i=1}^{5}{A}_{i}{e}^{-{\lambda\:}_{i}x},$$

69 $$\:{e}^{*}\left(x\right)=\sum\:_{i=1}^{5}{H}_{1i}{A}_{i}{e}^{-{\lambda\:}_{i}x},$$

70 $$\:{\omega\:}^{*}\left(x\right)=\sum\:_{i=1}^{5}{H}_{2i}{A}_{i}{e}^{-{\lambda\:}_{i}x},$$

71 $$\:{\phi\:}^{*}\left(x\right)=\sum\:_{i=1}^{5}{H}_{3i}{A}_{i}{e}^{-{\lambda\:}_{i}x},$$

72 $$\:{\theta\:}^{*}\left(x\right)=\sum\:_{i=1}^{5}{H}_{4i}{A}_{i}{e}^{-{\lambda\:}_{i}x},$$

73 $$\:{u}^{*}\left(x\right)=\sum\:_{i=1}^{5}{H}_{5i}{A}_{i}{e}^{-{\lambda\:}_{i}x},$$

74 $$\:{v}^{*}\left(x\right)=\sum\:_{i=1}^{5}{H}_{6i}{A}_{i}{e}^{-{\lambda\:}_{i}x},$$

75 $$\:{{\sigma\:}_{xx}}^{*}\left(x\right)=\sum\:_{i=1}^{5}{H}_{7i}{A}_{i}{e}^{-{\lambda\:}_{i}x},$$

76 $$\:{{\sigma\:}_{xy}}^{*}\left(x\right)=\sum\:_{i=1}^{5}{H}_{8i}{A}_{i}{e}^{-{\lambda\:}_{i}x},$$

77 $$\:{{\mu\:}_{xz}}^{*}\left(x\right)=\sum\:_{i=1}^{5}{H}_{9i}{A}_{i}{e}^{-{\lambda\:}_{i}x},$$

78 $$\:{{\mu\:}_{yz}}^{*}\left(x\right)=\sum\:_{i=1}^{5}{H}_{10i}{A}_{i}{e}^{-{\lambda\:}_{i}x}.$$

where

$$\:{H}_{1i}=\frac{{{\lambda\:}_{i}}^{2}-{\eta\:}_{9}}{{g}_{1}},\:{H}_{2i}=\frac{{{g}_{1}\left[\right({\lambda\:}_{i}}^{2}-{\eta\:}_{1}\left)\right({{\lambda\:}_{i}}^{2}-{\eta\:}_{6})-{\eta\:}_{7}{{(\lambda\:}_{i}}^{2}-{\mathrm{a}}^{2})}{{\upsilon\:}_{3}{\lambda\:}_{i}\left({{\lambda\:}_{i}}^{2}-{\eta\:}_{5}\right)\left({{\lambda\:}_{i}}^{2}-{\eta\:}_{6}\right)-{\eta\:}_{8}({{\lambda\:}_{i}}^{2}-{\eta\:}_{5})({{\lambda\:}_{i}}^{2}-{\mathrm{a}}^{2})}{H}_{1i},\:{H}_{3i}=\frac{{{\lambda\:}_{i}}^{2}-{\eta\:}_{5}}{{g}_{1}}{H}_{2i},$$

$$\:{H}_{4i}=\frac{{\eta\:}_{7}{H}_{1i}-{\eta\:}_{8}{H}_{3i}}{{{\lambda\:}_{i}}^{2}-{\eta\:}_{6}},\:{H}_{5i}=\frac{{iaH}_{3i}-{\lambda\:}_{i}{H}_{1i}}{{{\lambda\:}_{i}}^{2}-{\mathrm{a}}^{2}},\:{H}_{6i}=\frac{{H}_{1i}+{\lambda\:}_{i}{H}_{5i}}{ia}=\frac{{H}_{3i}-ia{H}_{5i}-{\lambda\:}_{i}}{{\lambda\:}_{i}},$$

$$\:{H}_{7i}=-{\beta\:}_{1}^{2}\left({\lambda\:}_{i}{H}_{5i}+{H}_{4i}\right)+ia{\delta\:}_{1}{H}_{6i},\:{H}_{8i}=-{\lambda\:}_{i}{H}_{6i}+ia{\delta\:}_{2}{H}_{5i}-2{H}_{2i},$$

$$\:{H}_{9i}=-{\lambda\:}_{i}{H}_{2i},\:{H}_{10i}=ia{H}_{2i},\:i=1,\:2,\:3\:,4,\:5.$$

## Boundary conditions

The arbitrary constants A_i​,_ $\:(i=\mathrm{1,2},\dots\:,5)$ appearing in the general solutions are determined by enforcing the boundary conditions at the free surface $\:x=0$.

$$\sigma_{xx}(0, y, t) = \sigma_{xy}(0, y, t) = \mu_{xz}(0, y, t) = \mu_{yz}(0, y, t) =0,$$

79 $$\:\theta\: (0, y, t) \:=Q, \mathrm{and}\:Q= \:{Q}_{o}{e}^{(\varpi\:t+iay)}.$$

The boundary conditions are chosen to reflect realistic constraints for displacement, temperature, stresses, and microrotation, ensuring mechanical and thermal equilibrium under combined thermal, magnetic, gravitational, and initial stress effects.

Substituting the general solutions into the boundary conditions at $\:x=0$ (Eq. 80) yields a linear system for the unknown constants A_i_:

80 $$\:\left[\begin{array}{c}{\:\:\mathrm{H}}_{41\:\:\:\:}\\\:{\:\mathrm{H}}_{71\:\:\:\:}\\\:{\mathrm{H}}_{81\:\:\:}\\\:{\mathrm{H}}_{91\:\:\:}\\\:{\mathrm{H}}_{101\:\:\:}\end{array}\:\:\:\begin{array}{c}{\:\:\:\mathrm{H}}_{42\:\:\:\:}\\\:{\:\:\:\mathrm{H}}_{72\:\:\:\:}\\\:{\:\:\:\mathrm{H}}_{82\:\:\:\:}\\\:{\:\:\mathrm{H}}_{92\:\:\:}\\\:{\:\mathrm{H}}_{102}\end{array}\begin{array}{c}\:\:\:\:{\:\:\:\mathrm{H}}_{43\:\:\:\:}\\\:\:\:\:\:\:\:\:{\mathrm{H}}_{73\:\:\:\:}\\\:\:\:\:\:{\mathrm{H}}_{83}\\\:\:\:\:\:\:{\mathrm{H}}_{93\:\:\:}\\\:\:\:\:\:{\mathrm{H}}_{103}\end{array}\:\:\:\:\:\:\begin{array}{c}\:{\:\:\:\mathrm{H}}_{44\:\:\:\:}\:\:\\\:\:\:{\mathrm{H}}_{74\:\:\:\:}\\\:\:\:{\:\:\mathrm{H}}_{84\:\:\:\:}\\\:{\:\:\mathrm{H}}_{94}\\\:\:\:\:{\mathrm{H}}_{104}\end{array}\begin{array}{c}{\:\:\:\:\:\:\:\mathrm{H}}_{45\:\:\:\:}\:\:\:\:\\\:\:\:{\:\mathrm{H}}_{75\:\:\:\:}\\\:\:\:\:\:{\mathrm{H}}_{85\:\:\:\:}\\\:{\:\mathrm{H}}_{95}\\\:\:\:{\mathrm{H}}_{105}\end{array}\right]\left[\begin{array}{c}{\mathrm{A}}_{1}\\\:{\mathrm{A}}_{2}\\\:{\mathrm{A}}_{3}\\\:{\mathrm{A}}_{4}\\\:{\mathrm{A}}_{5}\end{array}\right]=\left[\begin{array}{c}{Q}_{o}\\\:0\\\:0\\\:0\\\:0\end{array}\right].$$

To determine the constants $\:{A}_{1}$, $\:{A}_{2}$,$\:\:{A}_{3}$, $\:{A}_{4}$, and $\:{A}_{5}$Cramer’s method is applied to Eqs. (81),

81 $$\:{A}_{1}=\frac{\varDelta\:{A}_{1}}{\varDelta\:}, \:{A}_{2}=\frac{\varDelta\:{A}_{2}}{\varDelta\:}, \:{A}_{3}=\frac{\varDelta\:{A}_{3}}{\varDelta\:}, \:{A}_{4}=\frac{\varDelta\:{A}_{4}}{\varDelta\:}, \:{A}_{5}=\frac{\varDelta\:{A}_{5}}{\varDelta\:}.$$

## Validity of the model

When the thermoelastic wave (which is illustrated by gravity and initial stress is ignored, the magnetothermo-ealastic interactions in thermoelasticity theory in the framework of generalized micropolar thermoelastic medium is obtained and the results agree with Ezzat and Awad^21^. When the micropolar (which is illustrated by micropolar rotation $\:\omega\:(x,y,t)$ is ignored, the thermoelasticity theory is obtained and the results agree with Salah and Abd-Alla^5^.

## Numerical results and discussion

The analytical solutions derived in the previous sections are evaluated numerically to examine the effects of time, magnetic field, gravity, and initial stress parameters on wave propagation. Magnesium crystal material properties were adopted from Ezzat et al.^21^ for numerical evaluation.

**Table:** 

Unit	Symbol	Value	Unit	Symbol	Value
dyne. $\:{\boldsymbol{c}\boldsymbol{m}}^{-2}$	$\:\lambda\:$	$\:9.4{\:\times\:10}^{11}$	dyne. $\:{\boldsymbol{c}\boldsymbol{m}}^{-2}$	$\:\alpha\:$	$\:0.5{\:\times\:10}^{11}$
dyne. $\:{\boldsymbol{c}\boldsymbol{m}}^{-2}$	$\:\mu\:$	$\:4.5{\:\times\:10}^{11}$	dyne. $\:{\boldsymbol{c}\boldsymbol{m}}^{-2}$	$\:\upsilon\:+\beta\:$	$\:0.779{\:\times\:10}^{-4}$
$\:\boldsymbol{g}\boldsymbol{m}.{\boldsymbol{c}\boldsymbol{m}}^{-3}$	$\:\rho\:$	1.74	$\:\boldsymbol{c}\boldsymbol{a}\boldsymbol{l}.{\boldsymbol{g}\boldsymbol{m}}^{-1}.{\boldsymbol{c}}^{-1}$	$\:{C}_{e}$	$\:0.23$
$\:{\boldsymbol{o}}_{\boldsymbol{C}}$	$\:{T}_{o}$	23	$\:{\boldsymbol{c}\boldsymbol{m}}^{2}$	$\:j$	$\:0.2{\times\:10}^{-15}$
$\:{\boldsymbol{c}\boldsymbol{o}\boldsymbol{l}}^{2}.\:{\mathbf{d}\mathbf{y}\mathbf{n}\mathbf{e}}^{-2}.{\boldsymbol{c}\boldsymbol{m}}^{-2}$	$\:{\epsilon\:}_{o}$	$\:\frac{{10}^{-18}}{36\pi\:}$	$\:\mathbf{C}\mathbf{a}\mathbf{l}.\:{\boldsymbol{c}\boldsymbol{m}}^{-1}.{\boldsymbol{s}\boldsymbol{e}\boldsymbol{c}}^{-1}$	$\:{\sigma\:}_{o}$	$\:9.36{\times\:10}^{5}$
Cal. $\:{\boldsymbol{c}\boldsymbol{m}}^{-1}$.$\:{\boldsymbol{s}\boldsymbol{e}\boldsymbol{c}}^{-1}.{\boldsymbol{o}}_{\boldsymbol{C}}$	$\:K$	$\:0.6{\:\times\:10}^{-2}$	dyne. $\:{\boldsymbol{s}\boldsymbol{e}\boldsymbol{c}}^{2}.{\boldsymbol{c}\boldsymbol{o}\boldsymbol{l}}^{2}$	$\:{\mu\:}_{o}$	$\:4\pi\:{\times\:10}^{-2}$

**Fig. 2:**
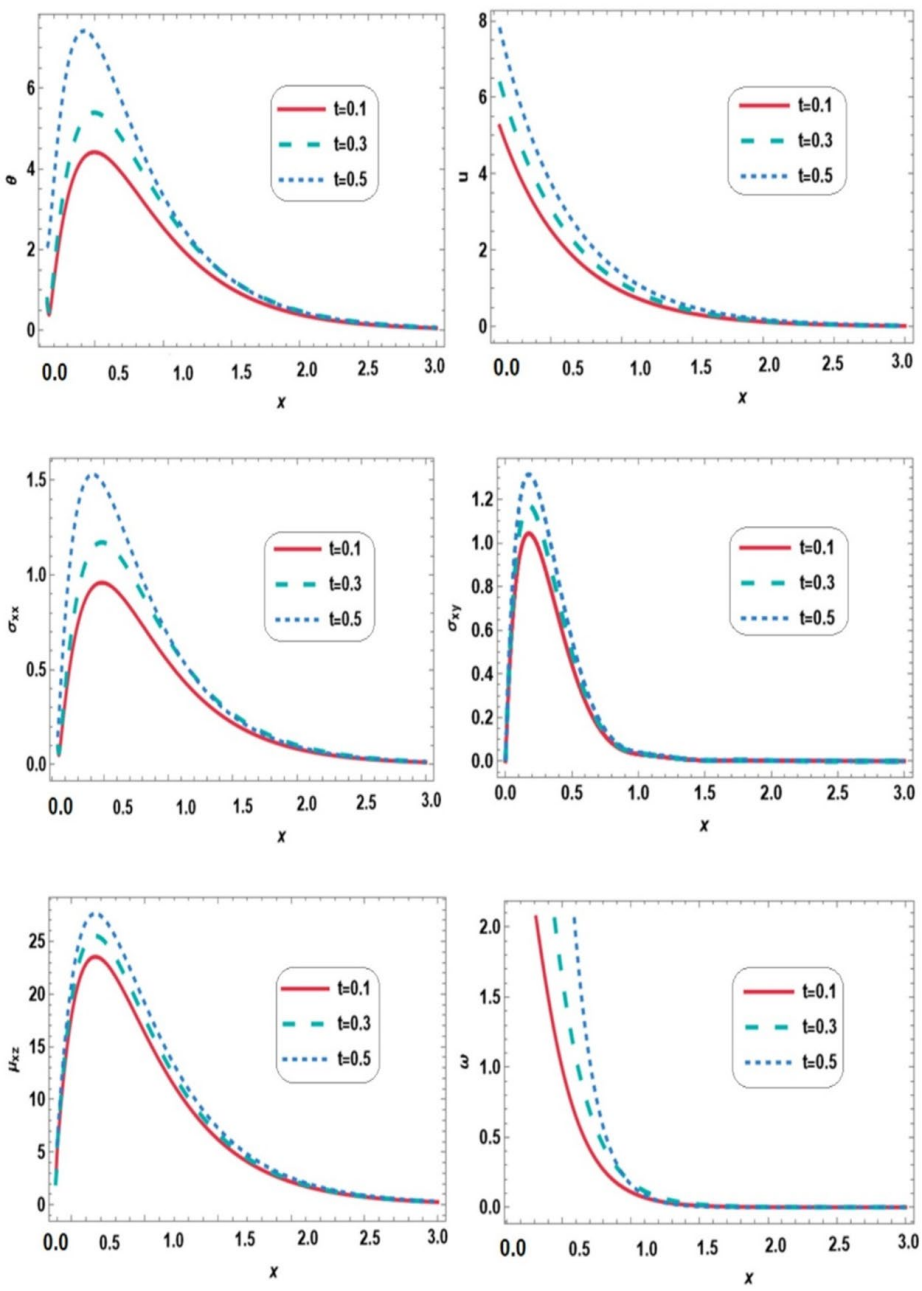
Variation of all physical quantities with distance$\:\:x$ at different times.

Figure 2 shows the variations of temperature $\:\theta\:$, displacement $\:u$, normal stress $\:{\sigma\:}_{xx}$, shear stress $\:{\sigma\:}_{xy}$, couple stress $\:{\mu\:}_{xz}$ and microrotation vector $\:\omega\:$ along the $\:x-$direction for different times ($\:t$ = 0.1, 0.3, and $\:0.5$) under thermal loading, magnetic field, gravity and initial stress. Within the interval $\:0\le\:x\le\:3$, increasing time leads to higher values of temperature, displacement, shear stress, couple stress, and microrotation. This behavior reflects the progressive diffusion of thermal energy into the medium, which induces thermal expansion and micro-rotational deformations. The mechanical response, represented by displacements and stresses, intensifies as the medium adjusts to the applied thermal load. The normal stress $\:{\sigma\:}_{xx}$ exhibits more pronounced variations compared to shear stress $\:{\sigma\:}_{xy}$ and couple stress $\:{\mu\:}_{xz}$​ indicating that the medium primarily accommodates the thermal effects through volumetric expansion rather than shear and rotational deformations. These results highlight the strong coupling between thermal, mechanical, and micropolar effects. Micropolar contributions are evident through the development of couple stresses and microrotation, which accommodate localized deformations not captured in classical thermoelasticity.

**Fig. 3:**
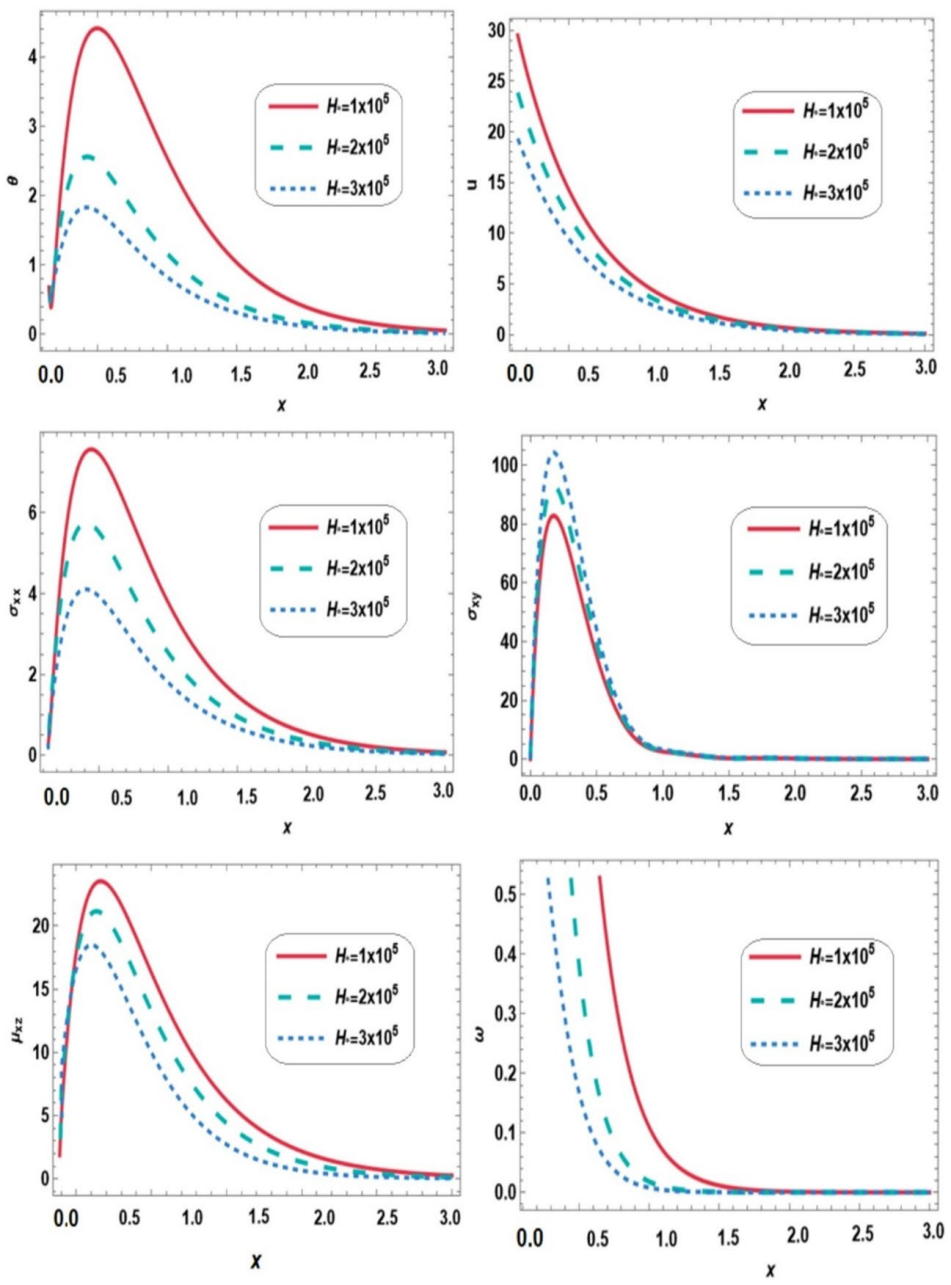
Effect of magnetic field on all physical quantities versus distance.

Figure 3 illustrates the variations of temperature $\:\theta\:$, displacement $\:u$, normal stress $\:{\sigma\:}_{xx}$, shear stress $\:{\sigma\:}_{xy}$, couple stress $\:{\mu\:}_{xz}$ and microrotation vector $\:\omega\:$ along the $\:x-$direction for different values of magnetic field ($\:{{{\rm\:H}}}_{o}=1\times\:{10}^{5},2\times\:{10}^{5},3\times\:{10}^{5}$) under the combined effects of thermal loading, gravity and initial stress. During the range $\:0\le\:x\le\:3$, the magnetic field alters wave propagation characteristics, leading to decreased temperature, displacement, normal stress, couple stress, and microrotation, while shear stress increases with increasing magnetic field strength. This behavior is attributed to the interaction between induced Lorentz forces and thermal expansion, which enhances shear and rotational deformation. Thus, rotational and shear mechanisms are activated to balance electromagnetic forces. These findings emphasize the strong coupling between thermal, mechanical, micropolar, and electromagnetic effects. The micropolar limit is approached when microrotation and couple stress become negligible, and the medium behaves classically.

**Fig. 4:**
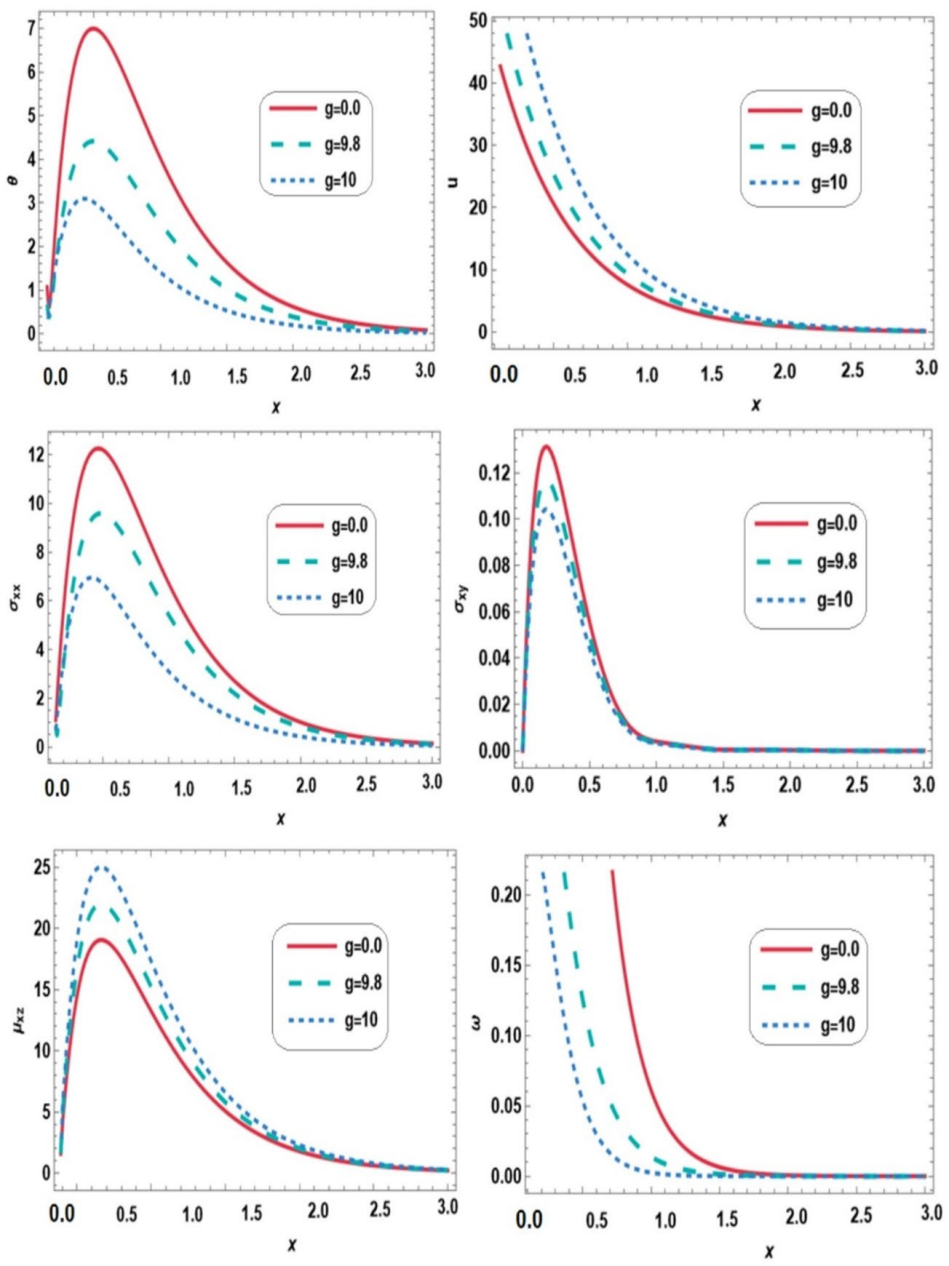
Effect of gravity on all physical quantities versus distance.

Figure 4 demonstrates the variations of temperature $\:\theta\:$, displacement $\:u$, normal stress $\:{\sigma\:}_{xx}$, shear stress $\:{\sigma\:}_{xy}$, couple stress $\:{\mu\:}_{xz}$ and microrotation vector $\:\omega\:$ along the $\:x-$direction for different values of gravity ($\:g=\mathrm{0,9.8,10}$) under the combined effects of thermal loading, a magnetic field and initial stress. The presence of gravity alters wave propagation in the range $\:0\le\:x\le\:3$, reducing temperature, normal stress, shear stress, and microrotation, while increasing displacement and couple stress. This reflects the influence of gravitational body forces opposing thermal expansion. Consequently, rotational and shear activities are reduced, while displacement and couple stresses increase to maintain mechanical equilibrium. These results highlight the role of gravity in modulating stress distribution and wave behavior through thermo-mechanical and micropolar coupling.

**Fig. 5:**
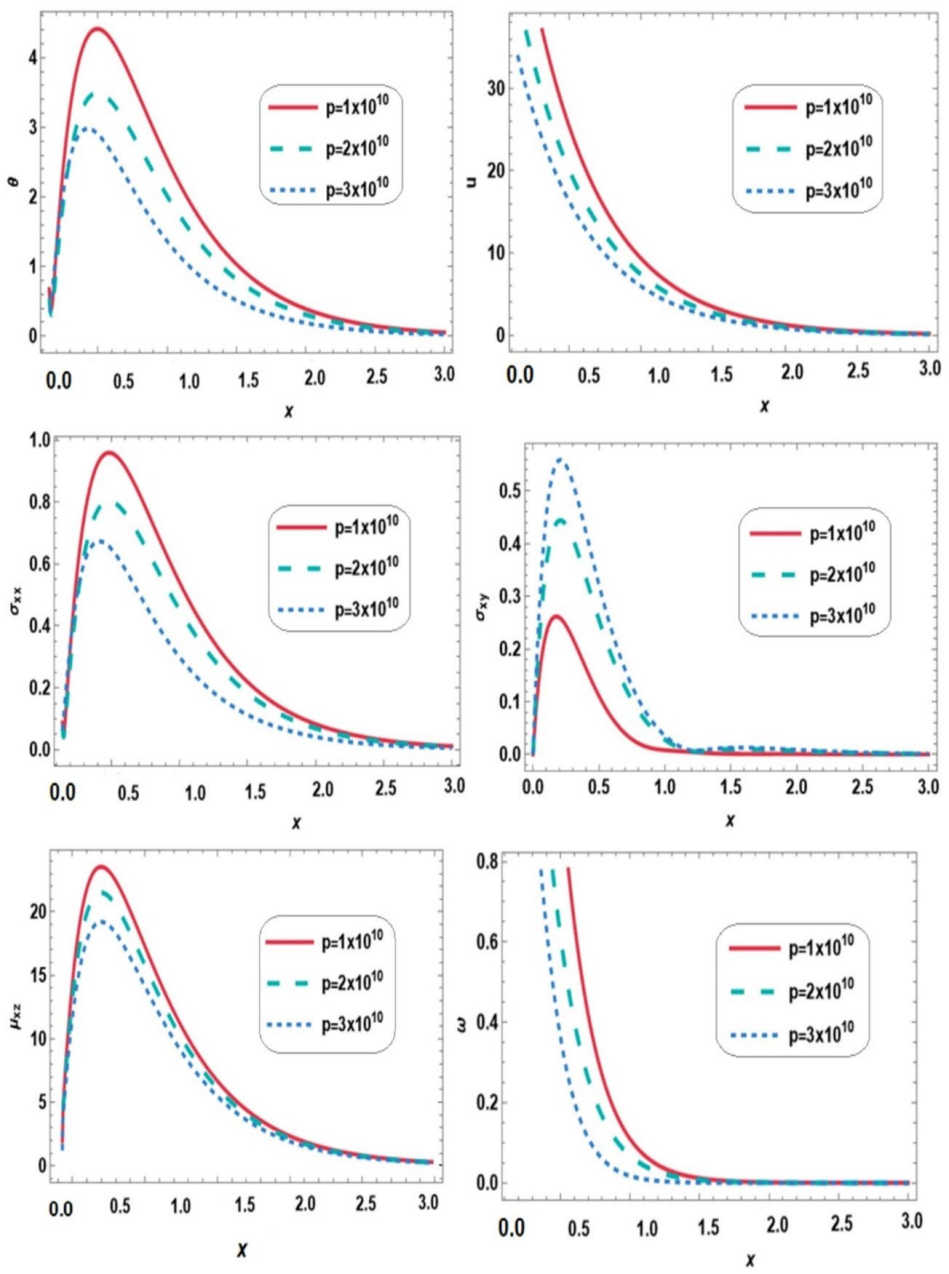
Effect of initial stress on all physical quantities versus distance.

Figure 5 displays the variations of temperature $\:\theta\:$, displacement $\:u$, normal stress $\:{\sigma\:}_{xx}$, shear stress $\:{\sigma\:}_{xy}$, couple stress $\:{\mu\:}_{xz}$ and microrotation vector $\:\omega\:$ along the $\:x-$direction for different values of initial stress ($\:p=1\times\:{10}^{10},2\times\:{10}^{10},3\times\:{10}^{10}$) under the combined effects of thermal loading and a magnetic field, and gravity. During the range $\:0\le\:x\le\:3$, The presence of initial stress alters wave propagation, leading to decreased temperature, displacement, normal stress, couple stress, and microrotation, while shear stress increases. Physically, initial stress acts as a pre-existing constraint that limits deformation and rotational motion, reducing expansion and microrotation while promoting shear to accommodate the imposed stress. This demonstrates that initial stress significantly influences wave propagation and stress distribution, with micropolar effects remaining important in capturing localized rotational behavior.

In summary, these results provide a mechanistic interpretation of how thermal, magnetic, gravitational, and initial stress effects influence wave propagation and stress distribution in a micropolar medium. The micropolar limit corresponds to cases where microrotation and couple stresses are negligible, yielding classical thermoelastic behavior. These insights confirm the physical consistency of the model and complement the trends observed in Figs. 2, 3, 4 and 5. The findings have practical relevance in the design and analysis of advanced smart materials, microstructured solids, thermal protection systems, magnetic sensing devices, and geophysical modeling of wave propagation in the Earth’s crust. The present results are consistent with previously published studies^9,13,16,17^, confirming the validity of the model and its agreement with established thermoelasticity theories.

## Limiting cases and model validation

To confirm the validity and generality of the proposed model, several limiting cases were examined:

1. Without Gravity: Neglecting gravitational forces reduces the model to a magneto-micropolar thermoelastic medium with initial stress, reproducing classical results^5,17,19,21^.
2. Without Initial Stress: Setting initial stress to zero simplifies the model to a magneto-micropolar thermoelastic medium with gravity and magnetic field, consistent with prior studies^5,16,18,21^.
3. Neglecting Micropolar Effects: Ignoring micropolar characteristics yields classical magneto-thermoelastic behavior, agreeing with traditional results^1–4,13^.

These comparisons demonstrate the mathematical correctness, physical consistency, and numerical reliability of the model. The analysis confirms that it accurately captures individual and combined effects of gravity, magnetic field, initial stress, and micropolarity, filling gaps in previous studies.

## Conclusion

Wave propagation in a generalized magneto-micropolar thermoelastic medium under the combined effects of gravity, magnetic field, and initial stress represents a complex multi-physics problem with important engineering and geophysical applications. This study quantifies the individual and combined influences of these factors on wave characteristics. Numerical simulations conducted in Mathematica provide insight into wave stability, attenuation, and dispersion under varying physical conditions. Overall, the results enhance the theoretical understanding of coupled thermoelastic systems and offer guidance for the design of materials and devices influenced by microstructural and thermal effects.

The main outcomes of the present study can be summarized as follows:

- The developed mathematical model captures the coupled interactions between thermal, magnetic, gravitational, and initial stress effects in a generalized magneto-micropolar thermoelastic medium.
- Thermal loading significantly influences temperature distribution and induces notable variations in displacement, stresses, couple stress, and microrotation due to strong thermo-mechanical coupling.
- The magnetic field modifies the mechanical behavior of the medium through electromagnetic interactions, leading to significant changes in stress fields, microrotation, and wave propagation characteristics.
- Gravitational forces introduce additional effects that alter wave propagation behavior, reducing temperature, normal stress, shear stress, and microrotation, while increasing displacement and couple stress to maintain mechanical equilibrium.
- Initial stress acts as a pre-stressing mechanism that constrains deformation and rotational motion, resulting in reduced displacement, temperature, normal stress, couple stress, and microrotation, while increasing shear stress.
- The numerical results demonstrate a strong sensitivity of wave propagation characteristics to variations in gravity and initial stress, highlighting their role in controlling the mechanical and micropolar response of the medium.
- All field variables satisfy the prescribed boundary conditions, confirming the correctness, consistency, and stability of the obtained solutions.
- The present theoretical framework has potential applications in the analysis and design of advanced smart materials, microstructured solids, and composite media subjected to combined thermal, magnetic, and gravitational environments.
- The results are particularly relevant to geophysical modeling of wave propagation in the Earth’s crust, thermal protection systems, magnetic sensing devices, and micro- and nano-scale engineering structures where initial stress and rotational effects are significant.

## Data Availability

The datasets used and/or analysed during the current study available from the corresponding author on reasonable request.

## Appendix A

$$\:{A}_{11}=-(-2{g}_{1}+{\eta\:}_{1}+{\eta\:}_{3}+{\eta\:}_{5}+{\eta\:}_{6}+{\eta\:}_{7}-{\eta\:}_{4}{\eta\:}_{8}+{\eta\:}_{9}-{\upsilon\:}_{2}{\upsilon\:}_{3}),$$

$$\:{A}_{22}=-(-{\eta\:}_{8}{\upsilon\:}_{2}-{\eta\:}_{4}{\eta\:}_{7}{\upsilon\:}_{3}),$$

$$\begin{aligned}&\:{A}_{33}=-(2{a}^{2}{g}_{1}+2{g}_{1}{\eta\:}_{1}+\varpi\:{\eta\:}_{2}-{\eta\:}_{1}{\eta\:}_{3}-{\eta\:}_{1}{\eta\:}_{5}-{\eta\:}_{3}{\eta\:}_{5}+2{g}_{1}{\eta\:}_{6}-{\eta\:}_{1}{\eta\:}_{6}-{\eta\:}_{3}{\eta\:}_{6}-{\eta\:}_{5}{\eta\:}_{6}\\&-{a}^{2}{\eta\:}_{7}+2{g}_{1}{\eta\:}_{7}-{\eta\:}_{3}{\eta\:}_{7}-{\eta\:}_{5}{\eta\:}_{7}+{a}^{2}{\eta\:}_{4}{\eta\:}_{8}+{\eta\:}_{1}{\eta\:}_{4}{\eta\:}_{8}+{\eta\:}_{4}{\eta\:}_{5}{\eta\:}_{8}+2{g}_{1}{\eta\:}_{9}-{\eta\:}_{1}{\eta\:}_{9}-{\eta\:}_{3}{\eta\:}_{9}\\&-{\eta\:}_{5}{\eta\:}_{9}-{\eta\:}_{6}{\eta\:}_{9}-{\eta\:}_{7}{\eta\:}_{9}+{\eta\:}_{4}{\eta\:}_{8}{\eta\:}_{9}+{\eta\:}_{5}{\upsilon\:}_{2}{\upsilon\:}_{3}+{\eta\:}_{6}{\upsilon\:}_{2}{\upsilon\:}_{3}+{\eta\:}_{9}{\upsilon\:}_{2}{\upsilon\:}_{3}),\end{aligned}$$

$$\:{A}_{44}=-({a}^{2}{\eta\:}_{8}{\upsilon\:}_{2}+{\eta\:}_{5}{\eta\:}_{8}{\upsilon\:}_{2}+{\eta\:}_{8}{\eta\:}_{9}{\upsilon\:}_{2}+{a}^{2}{\eta\:}_{4}{\eta\:}_{7}{\upsilon\:}_{3}+{\eta\:}_{4}{\eta\:}_{5}{\eta\:}_{7}{\upsilon\:}_{3}+{\eta\:}_{4}{\eta\:}_{7}{\eta\:}_{9}{\upsilon\:}_{3}),$$

$$\begin{aligned}&\:{A}_{55}=-(-2{a}^{2}{g}_{1}{\eta\:}_{1}+2\varpi\:{g}_{1}{\eta\:}_{2}-\varpi\:{\eta\:}_{2}{\eta\:}_{3}-\varpi\:{\eta\:}_{2}{\eta\:}_{5}+{\eta\:}_{1}{\eta\:}_{3}{\eta\:}_{5}-2{a}^{2}{g}_{1}{\eta\:}_{6}-2{g}_{1}{\eta\:}_{1}{\eta\:}_{6}\\&-\varpi\:{\eta\:}_{2}{\eta\:}_{6}+{\eta\:}_{1}{\eta\:}_{3}{\eta\:}_{6}+{\eta\:}_{1}{\eta\:}_{5}{\eta\:}_{6}+{\eta\:}_{3}{\eta\:}_{5}{\eta\:}_{6}-4{a}^{2}{g}_{1}{\eta\:}_{7}+{a}^{2}{\eta\:}_{3}{\eta\:}_{7}+{a}^{2}{\eta\:}_{5}{\eta\:}_{7}+{\eta\:}_{3}{\eta\:}_{5}{\eta\:}_{7}\\&-{a}^{2}{\eta\:}_{1}{\eta\:}_{4}{\eta\:}_{8}+\varpi\:{\eta\:}_{2}{\eta\:}_{4}{\eta\:}_{8}-{a}^{2}{\eta\:}_{4}{\eta\:}_{5}{\eta\:}_{8}-{\eta\:}_{1}{\eta\:}_{4}{\eta\:}_{5}{\eta\:}_{8}-2{a}^{2}{g}_{1}{\eta\:}_{9}-2{g}_{1}{\eta\:}_{1}{\eta\:}_{9}+{\eta\:}_{1}{\eta\:}_{3}{\eta\:}_{9}\\&+{\eta\:}_{1}{\eta\:}_{5}{\eta\:}_{9}+{\eta\:}_{3}{\eta\:}_{5}{\eta\:}_{9}-2{g}_{1}{\eta\:}_{6}{\eta\:}_{9}+{\eta\:}_{1}{\eta\:}_{6}{\eta\:}_{9}+{\eta\:}_{3}{\eta\:}_{6}{\eta\:}_{9}+{\eta\:}_{5}{\eta\:}_{6}{\eta\:}_{9}+{a}^{2}{\eta\:}_{7}{\eta\:}_{9}-2{g}_{1}{\eta\:}_{7}{\eta\:}_{9}\\&+{\eta\:}_{3}{\eta\:}_{7}{\eta\:}_{9}+{\eta\:}_{5}{\eta\:}_{7}{\eta\:}_{9}-{a}^{2}{\eta\:}_{4}{\eta\:}_{8}{\eta\:}_{9}-{\eta\:}_{1}{\eta\:}_{4}{\eta\:}_{8}{\eta\:}_{9}-{\eta\:}_{4}{\eta\:}_{5}{\eta\:}_{8}{\eta\:}_{9}-{\eta\:}_{5}{\eta\:}_{6}{\upsilon\:}_{2}{\upsilon\:}_{3}-{\eta\:}_{5}{\eta\:}_{9}{\upsilon\:}_{2}{\upsilon\:}_{3}\\&-{\eta\:}_{6}{\eta\:}_{9}{\upsilon\:}_{2}{\upsilon\:}_{3}),\end{aligned}$$

$$\:{A}_{66}=-(-{a}^{2}{\eta\:}_{5}{\eta\:}_{8}{\upsilon\:}_{2}-{a}^{2}{\eta\:}_{8}{\eta\:}_{9}{\upsilon\:}_{2}-{\eta\:}_{5}{\eta\:}_{8}{\eta\:}_{9}{\upsilon\:}_{2}-{a}^{2}{\eta\:}_{4}{\eta\:}_{5}{\eta\:}_{7}{\upsilon\:}_{3}-{a}^{2}{\eta\:}_{4}{\eta\:}_{7}{\eta\:}_{9}{\upsilon\:}_{3}-{\eta\:}_{4}{\eta\:}_{5}{\eta\:}_{7}{\eta\:}_{9}{\upsilon\:}_{3}),$$

$$\begin{aligned}&\:{A}_{77}=-(-2{a}^{2}\varpi\:{g}_{1}{\eta\:}_{2}+\varpi\:{\eta\:}_{2}{\eta\:}_{3}{\eta\:}_{5}+2{a}^{2}{g}_{1}{\eta\:}_{1}{\eta\:}_{6}-2\varpi\:{g}_{1}{\eta\:}_{2}{\eta\:}_{6}+\varpi\:{\eta\:}_{2}{\eta\:}_{3}{\eta\:}_{6}+\varpi\:{\eta\:}_{2}{\eta\:}_{5}{\eta\:}_{6}\\&-{\eta\:}_{1}{\eta\:}_{3}{\eta\:}_{5}{\eta\:}_{6}+2{a}^{4}{g}_{1}{\eta\:}_{7}-{a}^{2}{\eta\:}_{3}{\eta\:}_{5}{\eta\:}_{7}-{a}^{2}\varpi\:{\eta\:}_{2}{\eta\:}_{4}{\eta\:}_{8}+{a}^{2}{\eta\:}_{1}{\eta\:}_{4}{\eta\:}_{5}{\eta\:}_{8}-\varpi\:{\eta\:}_{2}{\eta\:}_{4}{\eta\:}_{5}{\eta\:}_{8}\\&+2{a}^{2}{g}_{1}{\eta\:}_{1}{\eta\:}_{9}-{\eta\:}_{1}{\eta\:}_{3}{\eta\:}_{5}{\eta\:}_{9}+2{a}^{2}{g}_{1}{\eta\:}_{6}{\eta\:}_{9}+2{g}_{1}{\eta\:}_{1}{\eta\:}_{6}{\eta\:}_{9}-{\eta\:}_{1}{\eta\:}_{3}{\eta\:}_{6}{\eta\:}_{9}-{\eta\:}_{1}{\eta\:}_{5}{\eta\:}_{6}{\eta\:}_{9}-{\eta\:}_{3}{\eta\:}_{5}{\eta\:}_{6}{\eta\:}_{9}\\&+4{a}^{2}{g}_{1}{\eta\:}_{7}{\eta\:}_{9}-{a}^{2}{\eta\:}_{3}{\eta\:}_{7}{\eta\:}_{9}-{a}^{2}{\eta\:}_{5}{\eta\:}_{7}{\eta\:}_{9}-{\eta\:}_{3}{\eta\:}_{5}{\eta\:}_{7}{\eta\:}_{9}+{a}^{2}{\eta\:}_{1}{\eta\:}_{4}{\eta\:}_{8}{\eta\:}_{9}+{a}^{2}{\eta\:}_{4}{\eta\:}_{5}{\eta\:}_{8}{\eta\:}_{9}\\&+{\eta\:}_{1}{\eta\:}_{4}{\eta\:}_{5}{\eta\:}_{8}{\eta\:}_{9}+{\eta\:}_{5}{\eta\:}_{6}{\eta\:}_{9}{\upsilon\:}_{2}{\upsilon\:}_{3}),\end{aligned}$$

$$\:{A}_{88}=-({a}^{2}{\eta\:}_{5}{\eta\:}_{8}{\eta\:}_{9}{\upsilon\:}_{2}+{a}^{2}{\eta\:}_{4}{\eta\:}_{5}{\eta\:}_{7}{\eta\:}_{9}{\upsilon\:}_{3}),$$

$$\begin{aligned}&\:{A}_{99}=-(2{a}^{2}\varpi\:{g}_{1}{\eta\:}_{2}{\eta\:}_{6}-\varpi\:{\eta\:}_{2}{\eta\:}_{3}{\eta\:}_{5}{\eta\:}_{6}+{a}^{2}\varpi\:{\eta\:}_{2}{\eta\:}_{4}{\eta\:}_{5}{\eta\:}_{8}-2{a}^{2}{g}_{1}{\eta\:}_{1}{\eta\:}_{6}{\eta\:}_{9}+{\eta\:}_{1}{\eta\:}_{3}{\eta\:}_{5}{\eta\:}_{6}{\eta\:}_{9}\\&-2{a}^{4}{g}_{1}{\eta\:}_{7}{\eta\:}_{9}+{a}^{2}{\eta\:}_{3}{\eta\:}_{5}{\eta\:}_{7}{\eta\:}_{9}-{a}^{2}{\eta\:}_{1}{\eta\:}_{4}{\eta\:}_{5}{\eta\:}_{8}{\eta\:}_{9})\end{aligned}.$$

## List of symbols

$$\:\rho\:$$: Material density
$$\:{\rho\:}_{e}$$: Charge density
T: Absolute temperature
$$\:{T}_{^\circ\:}$$: Reference temperature
$$\theta$$: $$\:\:=T-{T}_{^\circ\:}$$
$$\:u$$, $$\:v$$: Displacement components
$\:{{\upmu\:}}_{ij}$: Components of couple stress
$\:{\sigma\:}_{ij}$: Components of stress
$$\:{c}_{e}$$: Specific heat
$$\:\lambda\:$$, $$\:\mu\:$$: Lame’s constants
$\:{\alpha\:}_{t}$: Coefficient of linear thermal expansion
$$\:\gamma\:$$: $$\:=(3\lambda\:+$$2 $$\:\mu\:){\alpha\:}_{t}$$
$\:\alpha\:,\beta\:,\epsilon\:,\upsilon\:$: Micropolar elastic constants
$$\:j$$: Microinertia
$\:{\tau\:}_{o}$: Relaxation time parameter
k: Thermal conductivity
$$\:e$$: Dilatation
$\:{\delta\:}_{ij}$: Kronecker delta function
$$\:{e}_{ijk}$$: Permutation symbol
$$\:p$$: Initial stress
$\:{\omega\:}_{i}$: Components of micro-rotation
$\:{q}_{i}$: Components of heat flux
D: Electric displacement vector
E: Induced electric field vector
$$\:{\mu\:}_{o}$$: Magnetic permeability
$$\:{\sigma\:}_{o}$$: Electric conductivity
H: Magnetic intensity vector
h: Induced magnetic field vector
$\:{H}_{o}$: Initial magnetic field
$$\:{\epsilon\:}_{o}$$: Dielectric constant
J: Current density
g: Gravity
$$\:\phi\:$$: Thermal displacement
$$\:t$$: Time
$$\:a$$: Wave number
$$\:\varpi\:$$: Angular frequency
